# Shikonin Induced Program Cell Death through Generation of Reactive Oxygen Species in Renal Cancer Cells

**DOI:** 10.3390/antiox10111831

**Published:** 2021-11-18

**Authors:** Ming-Feng Tsai, Shih-Ming Chen, Ann-Zhi Ong, Yi-Hsuan Chung, Pei-Ni Chen, Yi-Hsien Hsieh, Yu-Ting Kang, Li-Sung Hsu

**Affiliations:** 1 Department of Nephrology, Antai Medical Care Cooperation Antai Tian-Sheng Memorial Hospital, Pingtung 92842, Taiwan; A097047@mail.tsmh.org.tw; 2 Bachelor Program in Health Care and Social Work for Indigenous Students, Providence University, Taichung 43301, Taiwan; big32762992@pu.edu.tw; 3 Institute of Medicine, Chung Shan Medical University, Taichung 40201, Taiwan; s0717011@gm.csmu.edu.tw (A.-Z.O.); s0717022@gm.csmu.edu.tw (Y.-H.C.); peini@csmu.edu.tw (P.-N.C.); hyhsien@csmu.edu.tw (Y.-H.H.); s0945011@gm.csmu.edu.tw (Y.-T.K.); 4 Clinical Laboratory, Chung Shan Medical University Hospital, Taichung 40201, Taiwan

**Keywords:** programed cell death, shikonin, reactive oxygen species, renal cell carcinoma

## Abstract

Shikonin mitigated tumor cell proliferation by elevating reactive oxygen species (ROS) levels. Herein, we investigated the effects of shikonin on renal cancer cell (RCC) cell proliferation. 3-(4,5-Dimethylthiazol-2-yl)-2,5-diphenyltetrazolium bromide (MTT) assay indicated that shikonin dose-dependently reduced the proliferation of Caki-1 and ACHN cells. Shikonin remarkably triggered necrosis and apoptosis in Caki-1 and ACHN cells in proportion to its concentration. Moreover, necrostatin-1 recovered cell viability in the presence of shikonin. Elevated ROS levels and mitochondrial dysfunction were also found in shikonin treatment groups. Pretreatment with N-acetyl cysteine remarkably mitigated shikonin-induced cell death and ROS generation. Western blot analysis revealed that shikonin reduced pro-PARP, pro-caspase-3, and Bcl-2 expression and increased cleavage PARP expression. Enhanced autophagy was also found in the shikonin-treated group as evidenced by acridine orange staining. Moreover, light chain 3B (LC3B)-II accumulation and enhanced p62 expression indicated that autophagy occurred in the shikonin-treated group. LC3B knockdown considerably recovered cell viability in the presence of shikonin. Shikonin treatment elevated p38 activity in a dose-dependent manner. In conclusion, our results revealed that shikonin triggered programmed cell death via the elevation of ROS level and p38 activity in different types of RCC cells. These findings suggested that shikonin may be a potential anti-RCC agent.

## 1. Introduction

Renal cell carcinoma (RCC) is a highly prevalent cancer all over the world and one of the top 10 cancers in the United States [1]. RCC can be divided into clear cell carcinoma (75–80%), papillary carcinoma (~15%), and chromophobe carcinoma (~5%) according to histopathological characteristics [2]. Surgical resection combined with chemotherapy is the standard treatment for RCC. However, the recurrence rate of metastatic RCC is over 40%. Therefore, searching for more effective drugs for RCC is an urgent issue.

Shikonin, a naphthoquinone derived from *Lithospermum erythrorhizon*, exerts broad functions [3]. Shikonin mitigates cell proliferation in various types of human cancer cell lines [4]. For example, shikonin remarkably elevated the percentage of necroptosis in glioma C6 and U87 MG cells in a dose-dependent manner, and this effect was diminished by necrostatin-1 (necroptosis inhibitor) [5]. Moreover, shikonin promoted cell death in triple-negative breast cancer MDA-MB-231 cells through the regulation of inosine 50-monophosphate dehydrogenase 2 [6]. Shikonin also suppressed nuclear-enriched abundant transcript 1 expression and mitigated cell proliferation in paclitaxel-resistant non-small cell lung cancer [7].

Reactive oxygen species (ROS), generated by the mitochondrial respiratory chain, and NADPH oxidase play critical roles in cancer formation and therapy [8]. Proper ROS level is involved in normal cell function; however, slightly increased ROS caused inactivation of the tumor to suppress gene and promoted oncogene activation in normal cells which may contribute to carcinogenesis [8]. Excessive ROS level triggers oxidative stress and induce apoptosis or necroptosis [9]. Accumulating reports have shown that shikonin promotes cell death through ROS generation. Shikonin remarkably elevated ROS level and extracellular-regulated kinase (ERK) activities and then triggered apoptosis in human 143B osteosarcoma cells [10]. Treatment with shikonin for 6 and 24 h increased ROS level and triggered necroptosis and apoptosis in human gastric AGS cancer cells, respectively [11]. Pretreatment with N-acetyl cysteine (NAC), an antioxidant molecule, reversed shikonin-induced ROS generation and cell death [11]. Shikonin promoted ROS production and then induced necroptosis in nasopharyngeal carcinoma cells [12] and glioma cells [13]. In addition, shikonin enhanced ROS generation through the regulation of NADPH oxidase activity in glioma cells [14].

Recently, using ingredients from Chinese traditional medicine as anti-RCC drugs has received intensive attention for their lower toxicity and fewer side effects. Emerging reports have been shown that shikonin exerts anti-tumor function in several human cancers. However, the impacts of shikonin on the cell death of RCC cells remain to be elucidated. In the present study, we investigated the anti-tumor effects of shikonin and gained insight into its possible molecular mechanisms in RCC cells using ACHN and Caki-1 cell lines. Herein, we first demonstrated that shikonin significantly induced multiple types of cell death in RCC cells via ROS-dependent manner.

## 2. Materials and Methods

### 2.1. Materials

Shikonin was purchased from ChemFaces (Wuhan, China). Thiazolyl blue tetrazolium bromide (MTT), 2′,7′-dichlorofluorescein diacetate (DCFH-DA), NAC, and other chemicals were obtained from Sigma-Aldrich (St. Louis, MI, USA). Anti-poly-ADP ribose polymerase was purchased from Cell Signaling Technology (No. 9542S, Beverly, MA, USA). Antibodies against ERK1/2 (ARG62350), phosphorylated ERK1/2 (ARG52277), and phosphorylated p38 (ARG51850) were obtained from Arigo Biolaboratories (Hsinchu City, Taiwan). Caspase 3 antibody (GTX110543) was purchased from GeneTex, Inc. (Hsinchu City, Taiwan). Light chain-3 (LC3)-II (NB 100-2220) antibody was obtained from Novus Biologicals (Centennial, CO, USA). Actin (6008-1-IG), Glyceraldehyde 3-phosphate dehydrogenase (GAPDH) (60004-1-Ig), and p38 (66234-1-Ig) were obtained from Proteintech Group, Inc. (Rosemont, IL, USA). Bcl-2 (TGABF-188784-25) was obtained from TrustGene Biotech Company (Taichung, Taiwan).

### 2.2. Cell Culture

RCC cell lines, ACHN (papillary carcinoma), and Caki-1 (clear cell carcinoma) were obtained from American Type Culture Collection (Manassas, VA, USA). The cells were kept in a minimum essential medium containing 10% fetal bovine serum and 10,000 U/mL penicillin and streptomycin and incubated at 37 °C with a humidified atmosphere of 5% CO_2_. For NAC groups, cells were pretreated with 5 mM NAC for 1 h before shikonin was added.

### 2.3. 3-(4,5-Dimethylthiazol-2-yl)-2,5-diphenyltetrazolium Bromide (MTT) Assay

Cells were seeded in a 24-well cultured plate at a density of 5 × 10^4^ cells/mL and then treated with 0, 4, 6, and 8 μM shikonin for 24 h. Afterward, the cells were incubated with a fresh medium with 0.5 mg/mL MTT for 2 h. The related cell proliferation was calculated by the absorbance value at 570 nm.

### 2.4. Detection of Reactive Oxygen Species (ROS) and Mitochondrial Membrane Potential

RCC cells were treated with 0, 4, 6, and 8 μM shikonin for 24 h and then stained with 10 μM DCFH-DA for 30 min. The cells were detached by trypsin treatment, washed with phosphate buffer saline (PBS) twice, and subjected to flow cytometry analysis with excitation and emission wavelengths of 485 and 535 nm, respectively.

Mitochondrial membrane potential was analyzed by JC-1 staining according to the manufacturer’s recommendation. Green/red fluorescence was detected by flow cytometry.

### 2.5. Propidium Iodide (PI)/Annexin V Double Staining

The cells were treated with 0, 4, 6, and 8 μM shikonin for 24 h, then detached by trypsin and stained with Dead Cell Apoptosis Kit according to the manufacturer’s recommendation (No. V13241, Thermo Fisher Scientific, Inc., Waltham, MA, USA). The signals were detected by measuring the fluorescence emission at 530 and 575 nm using 488 nm excitation by flow cytometry.

### 2.6. Acridine Orange Staining

For acridine orange staining, cells were seeded in 3 cm culture dishes at a density of 3 × 10^5^. After being treated with 0 or 6 μM shikonin for 24 h, cells were incubated with a medium containing 10 μg/mL acridine orange for 30 min in the dark. Cells were washed with PBS three times, and the signals were captured under a fluorescent microscope.

### 2.7. Knockdown of LC3B

For knockdown of LC3B, the short-hairpin RNA lentiviral particle (shRNA; No. TRCN0000155417) with target sequences of CCTGACCATGTCAACATGAGT was purchased from the National RNAi Core Facility of Academia Sinica (Taipei, Taiwan). ACHN and Caki-1 cells were seeded at a density of 1 × 10^5^/well in a 6-well plate. The cells were transfected with a 2 mL medium containing 100 μL virus particle for 5 min at 37 °C and then added 1 mL fresh medium containing protamine sulfate to a final concentration of 8 μg/mL. Twenty-four h post-transfection, the medium was replaced with a fresh medium containing 2 μg/mL puromycin and incubated for an additional 48 h. The cell lysate was collected and subjected to Western blot analysis to confirm the knockdown effects. The luciferase lentiviral particle (No. TRCN0000072246) with the target sequence of CAAATCACAGAATCGTCGTAT was used as a scramble control.

### 2.8. Western Blot Analysis

The cells were treated with the indicated concentrations of shikonin with or without 5 mM NAC pretreatment for 24 h. Proteins were extracted by radioimmunoprecipitation assay buffer containing proteinase inhibitors, and their concentrations were measured by the Bio-Rad Protein Assay Kit (Bio-Rad Laboratories, Inc., Hercules, CA, USA). A total of 30 μg proteins was separated by 4–20% sodium dodecyl sulfate–polyacrylamide gel electrophoresis and then electro-transferred into polyvinylidene fluoride membrane (Merk Millipore, Burlington, MA, USA). The membrane was blocked by PBS with 5% non-fat milk for 1 h at room temperature. Then, the membrane was washed with PBS thrice and incubated in PBS containing the indicated antibody overnight at 4 °C. Then, the membrane was washed with PBS three times and immersed in PBS containing horseradish peroxidase-conjugated anti-rabbit or anti-mouse secondary antibodies at room temperature for 1 h. The membrane was washed with PBS, and a positive signal was detected by an enhanced chemiluminescent kit (Merk Millipore, Burlington, MA, USA). Images were captured and analyzed by Image J software (National Institutes of Health, Bethesda, MD, USA). β-actin or GADPH signal was used as the internal control.

### 2.9. Statistical Analysis

All data were obtained from at least three independent experiments and presented as mean ± standard deviation. Statistical comparison between multiple groups was performed using one-way ANOVA followed by Dunnett’s post hoc test or Student *t*-test using GraphPad Prism software (San Diego, CA, USA). *p* < 0.05 was considered significant difference.

## 3. Results

### 3.1. Shikonin Mitigated Cell Proliferation of Renal Cancer Cell (RCC) through Induction of Apoptosis and Necrosis

**Figure 1:**
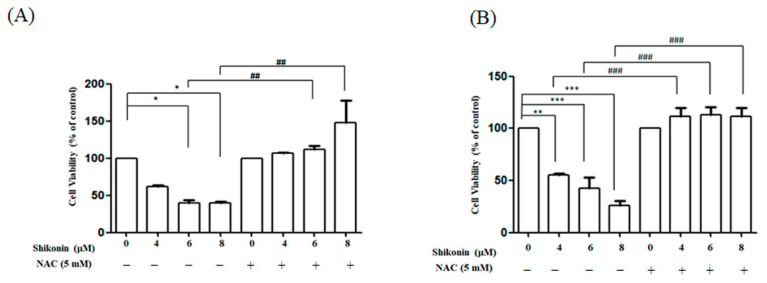
Shikonin dose-dependent mitigated cell viability in renal cancer cell (RCC) cells, and N-acetyl cysteine (NAC) reversed the phenomenon. 3-(4,5-Dimethylthiazol-2-yl)-2,5-diphenyltetrazolium bromide (MTT) assays of (**A**) ACHN and (**B**) Caki-1 cells treated with shikonin for 24 h with or without pretreatment with 5 mM NAC. Data represent means ± standard deviation from at least three independent experiences. * *p* < 0.05, ** *p* < 0.01 and *** *p* < 0.001 compared with the control group. ## *p* < 0.01 and ### *p* < 0.001 compared with the NAC pretreated group.

MTT assay was conducted to assess the effects of shikonin on RCC cell proliferation. Cell viability decreased to 62.1%, 39.8%, and 39.5% in ACHN cells and 51.9%, 49.1%, and 29.1% in Caki-1 cells after exposure to 4, 6, and 8 μM shikonin for 24 h, respectively. Our result indicated that shikonin dose-dependently repressed the viability of RCC cells (Figure 1). In addition, pretreatment with NAC recovered the cell viability to a level close to that of the control group.

**Figure 2:**
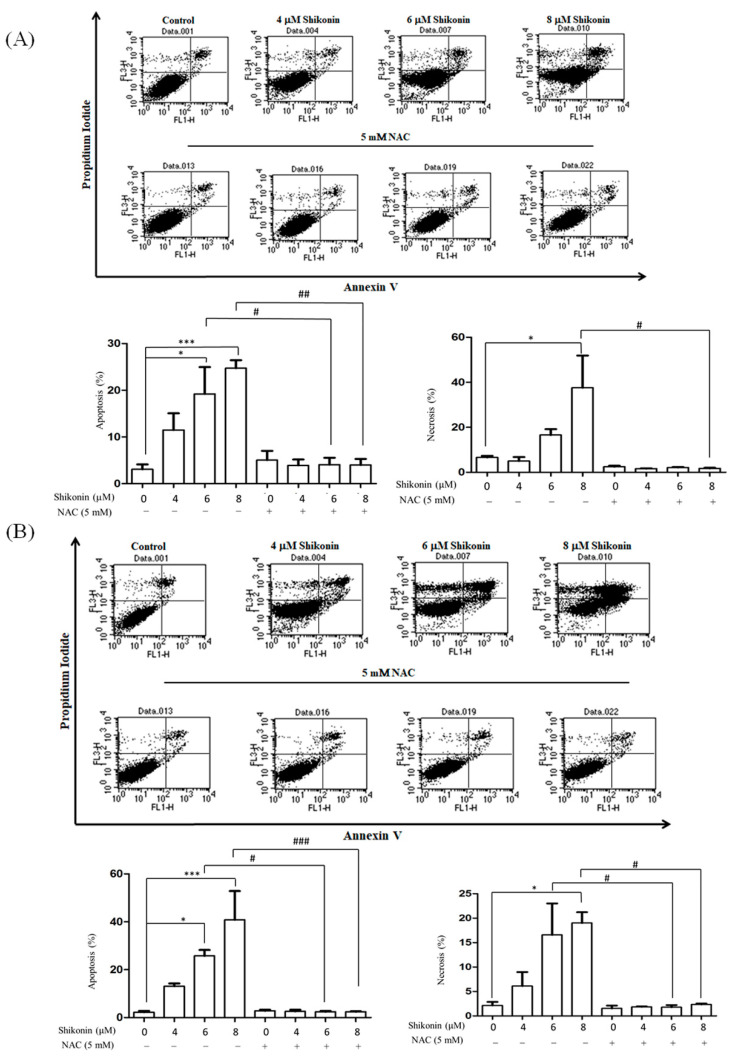
Shikonin induced apoptosis and necrosis in RCC cells. PI/annexin V double staining of (**A**) ACHN and (**B**) Caki-1 cells treated with shikonin for 24 h with or without pretreatment with NAC. Data represent means ± standard deviation from at least three independent experiences. * *p* < 0.05 and *** *p* < 0.001 compared with the control group. # *p* < 0.05, ## *p* < 0.01, and ### *p* < 0.001 compared with the NAC pretreated group.

Annexin V and PI double staining was conducted to further identify the cell death modes induced by shikonin. The apoptosis (annexin V positive) rates were 11.5%, 19.2%, and 24.7% and the necrosis (PI-positive, annexin V-negative) rates were 2.0%, 16.6%, and 37.6% in CaKi-1 cells in the presence of 4, 6, and 8 μM shikonin, respectively. In comparison, the apoptosis rates were 13.1%, 26.5%, and 40.8% and the necrosis rates were 6.4%, 18.0%, and 19.5% in ACHN cells in response to 4, 6, and 8 μM shikonin treatment, respectively (Figure 2). NAC also blocked the shikonin-induced apoptosis and necrosis.

**Figure 3:**
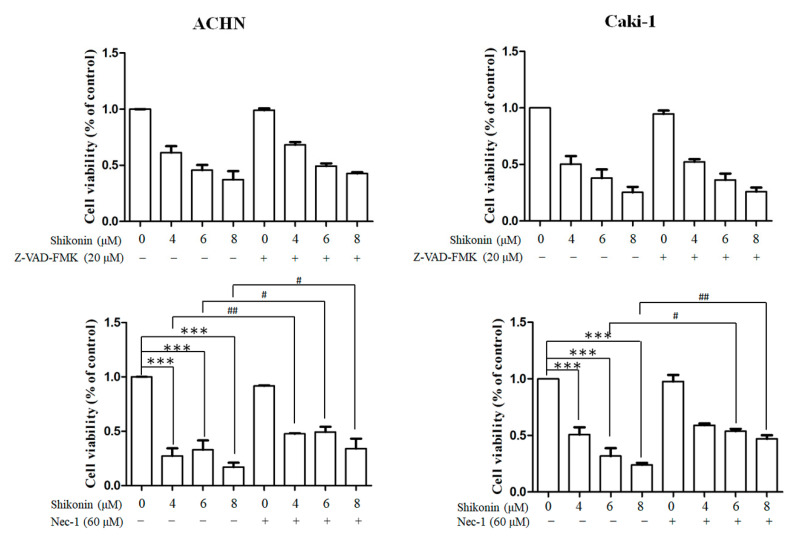
Necrostatin-1 but not Z-VAD-FMK reversed shikonin-induced cell death. ACHN (**left panel**) and Caki-1 (**right panel**) were treated with shikonin with or without Z-VAD-FMK (**upper panel**) or necrostatin-1 (Nec-1; **lower panel**). Data represent means ± standard deviation from at least three independent experiences. *** *p* < 0.001 compared with the control group. # *p* < 0.05 and ## *p* < 0.01 compared with the Nec-1 pretreated group.

In addition, we conducted inhibitors for apoptosis and necrosis to confirm the effects of shikonin. No overt alteration in cell viability was found in RCC cells pretreated with caspase inhibitor, Z-VAD-FMK. Cell viability was remarkably elevated in Caki-1 and ACHN cells pretreated with necrosis inhibitor, necrostatin-1, in the presence of 6 and 8 μM shikonin (Figure 3).

### 3.2. Shikonin Enhanced ROS Level and Triggered Mitochondrial Dysfunction

**Figure 4:**
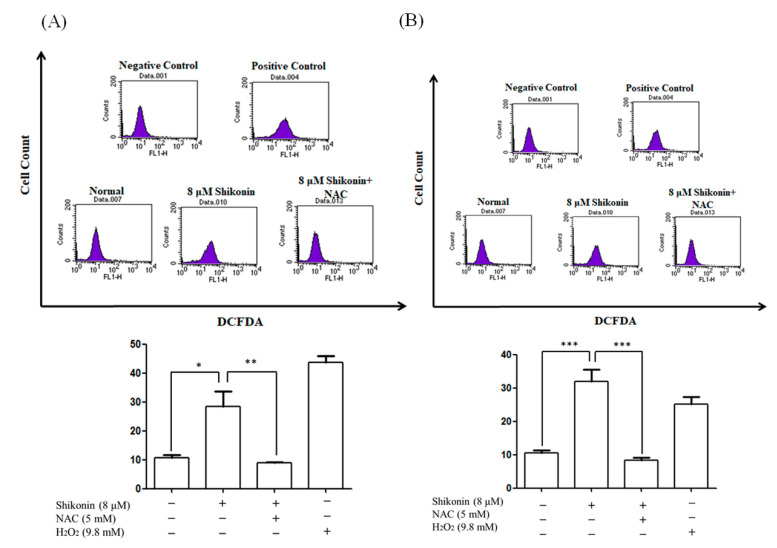
Shikonin enhanced reactive oxygen species (ROS) level in RCC cells. (**A**) ACHN and (**B**) Caki-1 cells were treated with 8 μM shikonin for 24 h with or without pretreatment with NAC. The cells were stained with DCFH-DA and analyzed by flow cytometry. H_2_O_2_ was used as the positive control. Data represent means ± standard deviation from at least three independent experiences. * *p* < 0.05, ** *p* < 0.01, and *** *p* < 0.001.

Previous reports demonstrated that shikonin enhances ROS production in several cancers [4]. DCFH-DA staining was performed to determine whether shikonin triggers ROS generation. As shown in Figure 4, the mean fluorescence intensity increased to 28.4% and 31.9% in response to 8 μM shikonin treatment compared with 10.8% and 10.5% in the control groups in ACHN and Caki-1 cells, respectively. Pre-treated with NAC also reduced the mean fluorescence intensity to 8.8% in ACHN and 8.3% in Caki-1, respectively.

**Figure 5:**
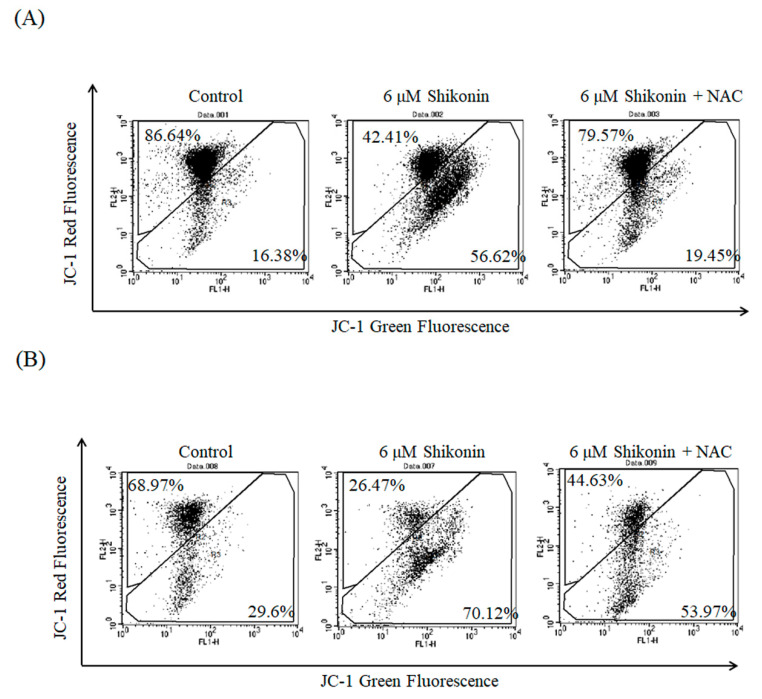
Shikonin disrupted mitochondrial membrane potential in RCC cells. ACHN (**A**) and Caki-1 (**B**) cells were treated with shikonin for 24 h with or without pretreatment with NAC and then stained with JC-1. Data represent the flow cytometry analysis of JC-1 staining.

We assumed that ROS generation led to the loss of mitochondrial membrane potential; thus, we conducted JC-1 staining to verify our assumption. In normal conditions, JC-1 forms a dimer with red color whereas JC-1 becomes a green monomer in the presence of disruption of mitochondrial membrane potential. As shown in Figure 5, the green fluorescence obviously increased (56.6% in ACHN cells and 70.1% in Caki-1 cells) under 6 μM shikonin treatment for 24 h compared with the control (16.4% in ACHN cells and 29.6% in Caki-1 cells). NAC pretreatment reduced the green fluorescence to 19.5% in ACHN cells and 54.0% in Caki-1 cells, respectively.

### 3.3. Shikonin Affected the Expression of Apoptosis-Related Proteins

**Figure 6:**
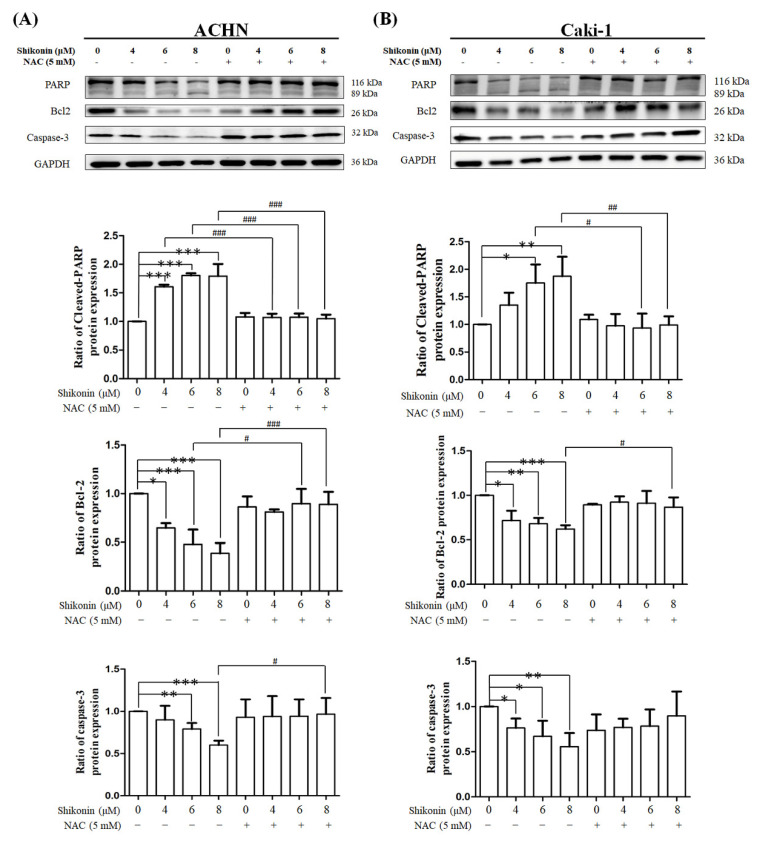
Shikonin affected the expression of apoptosis-related proteins. (**A**) ACHN and (**B**) Caki-1 cells were treated with 0, 4, 6, and 8 μM shikonin with or without pretreatment with NAC. The cell lysates were analyzed by Western blot using the indicated primary antibody. Data represent means ± standard deviation from at least three independent experiences. * *p* < 0.05, ** *p* < 0.01, and *** *p* < 0.001 compared with the control group. # *p* < 0.05, ## *p* < 0.01, and ### *p* < 0.001 compared with the NAC pretreated group. Glyceraldehyde 3-phosphate dehydrogenase (GAPDH) was used as the internal control.

Western blot analysis was conducted to evaluate whether shikonin regulates the expression of apoptosis-related proteins. Shikonin dose-dependently decreased pro-PARP expression and increased cleavage PARP expression in ACHN and Caki-1 cells (Figure 6). In addition, pro-caspase-3 and Bcl-2 expression levels were substantially decreased in ACHN and Caki-1 cells in proportion to shikonin concentration. Similarly, NAC pretreatment recovered the shikonin-induced changes.

### 3.4. Shikonin Promoted Autophagy in RCC Cells

**Figure 7:**
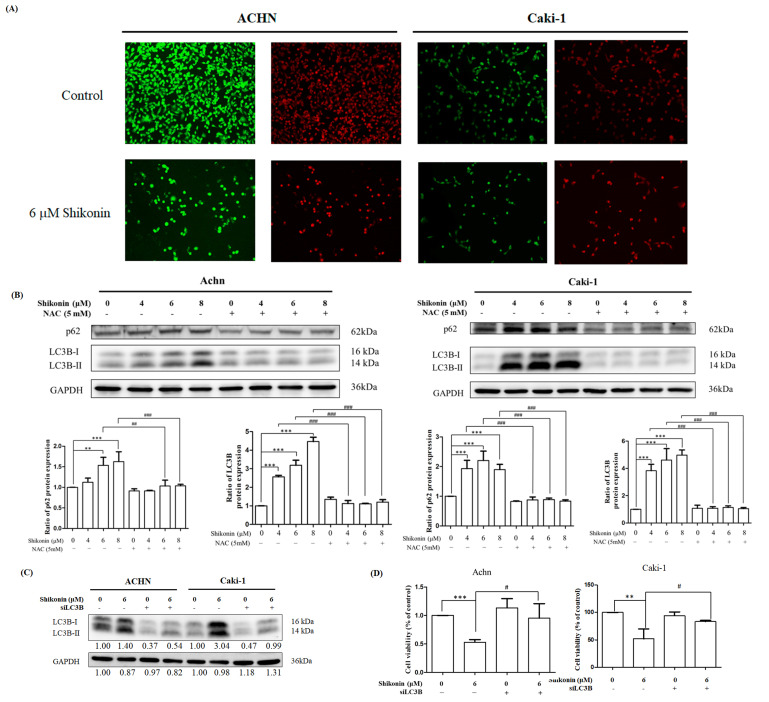
Shikonin induced autophagy in RCC cells. (**A**) ACHN (right panel) and Caki-1 (left panel) cells were treated with 0 or 6 μM shikonin for 24 h and subjected to acridine orange staining and the images were captured under a fluorescence microscope. (**B**) ACHN and Caki-1 cells were treated with 0, 4, 6, and 8 μM shikonin with or without NAC pretreatment. The cell lysates were analyzed by Western blot using LC3B and p62 antibodies. GAPDH was used as the loading control. Data represent means ± standard deviation from at least three independent experiences. ** *p* < 0.01 and *** *p* < 0.001 compared with the control group. # *p*< 0.05, ## *p* < 0.01 and ### *p* < 0.001 compared with the NAC pretreated group. GAPDH was used as the internal control. (**C**) ACHN and Caki-1 cells were treated with 6 μM shikonin in the presence or absence of LC3B-II siRNA (siLC3B) for 24 h. The cell lysates were analyzed by Western blot using LC3B antibody. (**D**) ACHN and Caki-1 cells were treated with 6 μM shikonin in the presence or absence of siLC3B for 24 h. Cell viability was measured by MTT assay. Data represent means ± standard deviation from at least three independent experiences. ** *p* < 0.01 and *** *p* < 0.001 compared with the control group. # *p* < 0.05 compared with the siLC3B group.

To investigate whether shikonin triggered autophagy, acridine orange staining was conducted. The cell viability was obviously decreased in the presence of 6 μM shikonin (green fluorescence) in ACHN and Caki-1 cells. On the other hand, higher intensity of red fluorescence was found in the presence of 6 μM shikonin (Figure 7A). These results suggested that shikonin promoted autophagy in RCC.

Western blot analyses of p62 and LC3 were also performed to determine whether shikonin affects the expression of autophagy-related proteins. LC3B-II and p62 expression levels were considerably elevated in proportion to shikonin concentration. Similarly, NAC pretreatment diminished the shikonin-induced changes in LC3B-II and p62 expression (Figure 7B).

LC3B was knocked down by shRNA to further investigate the effects of autophagy on shikonin-induced cell death. LC3B-II accumulation was obviously mitigated in the LC3B knockdown group after shikonin treatment (Figure 7C). As shown in Figure 7D, LC3B knockdown remarkably increased cell viability (95.3% in ACHN cells and 83.8% in Caki-1 cells) compared with 6 μM shikonin treatment (53.0% in ACHN cells and 51.7% in Caki-1 cells).

### 3.5. Effect of Shikonin on ERK and p38 Activities

**Figure 8:**
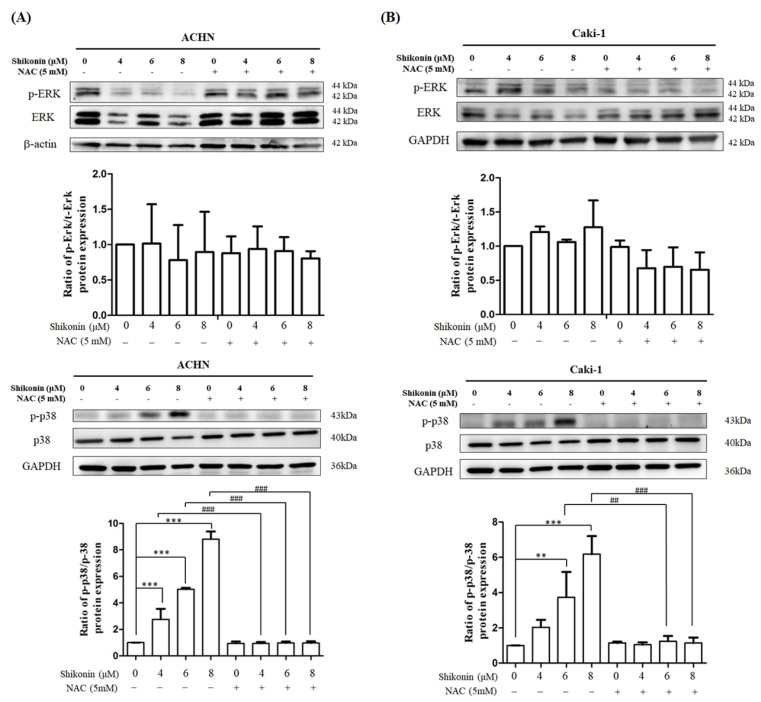
Shikonin promoted p38 activity but not ERK activity. (**A**) ACHN and (**B**) Caki-1 cells were treated with 0, 4, 6, and 8 μM shikonin with or without NAC pretreatment. The cell lysates were analyzed by Western blot using ERK or p38 antibodies. Data represent means ± standard deviation from at least three independent experiences. ** *p* < 0.01 and *** *p* < 0.001 compared with the control group. ## *p* < 0.01 and ### *p* < 0.001 compared with the NAC pretreated group. GAPDH was used as the internal control.

Western blot analysis was performed to measure the effects of shikonin on ERK and p38 activities. No change in ERK activity was found in the presence of 4, 6, and 8 μM shikonin. In comparison, phosphorylated p38 was remarkably enhanced in ACHN and Caki-1 cells in proportion to shikonin concentration (Figure 8).

## 4. Discussion

RCC is one of the top 10 leading causes of cancer-related deaths worldwide. Mortality is increased in advanced RCC stages because of chemotherapy resistance and distal metastasis. Natural compounds from vegetables or Chinese traditional medicine have been shown to induce cell death in human cancers through multiple pathways. Shikonin, a naphthoquinone pigment isolated from *L. erythrorhizon*, suppressed cell proliferation in osteosarcoma [10] and gastric cancer [11]. However, the molecular mechanisms of shikonin-induced cell death remain to be elucidated in RCC. In the present study, we showed that shikonin obviously diminished RCC cell proliferation by triggering apoptosis, necroptosis, and autophagy. Blocking ROS production recovered shikonin-induced cell death in RCC. In addition, alteration in p38 activities was found in response to shikonin treatment.

ROS are produced from two major pathways: (1) as byproducts of the electron transport chain in the mitochondria and (2) NADPH oxidases [8]. Increased ROS levels lead to tumorigenesis in normal cells [8]. However, documents have been well established that extracts from traditional Chinese medicine induce cancer cell death by extensive ROS generation in several cancer cells [15]. Shikonin induced necrosis after 6 h of treatment and apoptosis after 24 h of treatment through the elevation of ROS levels in human AGS gastric cells [11]. Pretreated with the antioxidant agent, NAC, reversed the shikonin-induced increase in ROS level and cell death [11]. Similarly, shikonin also promoted ROS generation in nasopharyngeal carcinoma [12], colon cancer [16], and osteosarcoma cells [10]. In line with these observations, shikonin considerably increased ROS levels and caused mitochondrial dysfunction in RCC cells pretreated with NAC.

Apoptosis is a major type of programmed cell death. Apoptosis is characterized as the formation of apoptotic bodies and DNA fragmentation [17]. In colorectal cancer SW-620 cells, shikonin promoted galectin-1 dimerization and triggered apoptosis in a ROS-dependent manner [18]. In addition, the forced expression of peroxiredoxin V mitigated shikonin-induced apoptosis in colon cancer cells [19]. Shikonin enhanced apoptosis in chronic myeloid leukemia cells through AKT inhibition [20]. In the present study, we demonstrated that shikonin considerably triggered apoptosis in a concentration-dependent manner as evidenced by PI/annexin V double staining analysis. Moreover, shikonin also increased cleavage PARP and Bcl-2 expression and decreased pro-caspase 3 expression. However, cell viability was recovered by pretreatment with NAC but not caspase inhibitor, Z-VAD-FMK. Consistently, Z-VAD-FMK had no effects on shikonin-induced cell death in glioma [5], non-small cell lung cancer cells [21], and osteosarcoma cells [22]. Together, our results suggested that shikonin-induced apoptosis in RCC cells via ROS generation.

Necrosis, another major type of programmed cell death, was also involved in shikonin-induced cell death. Shikonin triggered necrosis in osteosarcoma [10], gastric cancer [11], and nasopharyngeal carcinoma cells [23] through the promotion of ROS generation. Shikonin induced necroptosis in prostate cancer and glioma cell as evidenced by the enhanced phosphorylation of receptor interacting protein kinase 1, and this effect was blocked by necrostatin-1 pretreatment [13,24]. In line with these observations, we demonstrated that shikonin substantially induced necrosis as evidenced by the increased PI-positive population in the double staining analysis, and pretreatment with NAC and necrostatin-1 recovered cell viability in the presence of shikonin. Collectedly, shikonin enhanced necrosis in RCC in a ROS-dependent manner.

Autophagy plays a critical role in maintaining cell homeostasis and metabolic balance through delivering damaged organelles and useless proteins to the lysosome for degradation [25]. Autophagy exerted anti-death or pro-death in distinct cancer cells in response to different stimuli. Gong et al. demonstrated that shikonin promoted ROS generation, which leads to ERK activation and rendered hepatocellular carcinoma cells to undergo autophagy in cultured cell lines and tumor xenograft model [26]. Shikonin increased LC3B-II and reduced p62, phosphorylated phosphoinositide 3-kinase, and AKT, which then triggered autophagy in XPC-3 human pancreatic cancer cells [27]. In A375 human melanoma cells, shikonin induced protective autophagy effects through ROS and p38 pathway [28]. Autophagy inhibition by 3-methyladenine enhanced shikonin-induced apoptosis in melanoma A375 cells [28]. Shikonin promoted galectin-1 dimer, then activated Jun N-terminal protein kinase (JNK), and eventually caused apoptosis and autophagy in colorectal cancer [18]. In line with previous observations, shikonin also caused autophagy as evidenced by increased acridine orange staining and LC3B-II accumulation in this presentation. However, shikonin enhanced rather than decreased p62 expression in this study. Consistently, chalcone flavokawain B induced p62 expression and autophagy via a ROS-dependent manner in human melanoma cells [29]. Pretreatment with NAC diminished shikonin-induced autophagy-related phenomena. Moreover, LC3 knockdown suppressed shikonin-induced cell death in RCC cells. Collectedly, our findings indicated that shikonin triggered autophagy in RCC cells through ROS generation.

Mitogen-activated protein kinase (MAPK) family, including ERK, p38, and JNK, play a critical role in cell proliferation, apoptosis, and differentiation [30]. Reports have shown that shikonin manipulates MAPK signals to cause cell death. In osteosarcoma 143B cells, shikonin promoted apoptosis through increased ERK activity [10]. On the other hand, shikonin repressed ERK activity and then triggered cell death in lung, pancreatic, and breast cancer cells [31]. However, in this study, no overt alteration in ERK activity was found in response to shikonin treatment. Emerging documents also have shown that p38 is involved in shikonin-induced apoptosis. In human AGS gastric cancer cells, shikonin elevated p38 activity, reduced AKT activity, and then promoted apoptosis [11]. Moreover, shikonin also triggered apoptosis by enhancing p38 activity in human melanoma A375 [28] and human leukemia NB4 cells [32]. In line with these observations, our results demonstrated that shikonin activated p38 activity and participated in apoptosis. Collectively, our data revealed that shikonin induced p38 activity but not ERK activity and rendered RCC cells to undergo apoptosis.

## 5. Conclusions

Herein, we highlighted the molecular mechanisms of shikonin-induced programmed cell death in RCC. Shikonin significantly promoted ROS generation, triggered mitochondrial dysfunction, and finally induced programmed cell death. NAC, an antioxidant agent, obviously reversed shikonin-induced cell death. In summary, our findings provided the first pieces of evidence that shikonin mitigated the proliferation of RCC and could be a promising anti-renal cancer agent.

## Data Availability

The data is contained within the article.
